# Beyond vessels: unraveling the impact of VEGFs on neuronal functions and structure

**DOI:** 10.1186/s12929-025-01128-8

**Published:** 2025-03-06

**Authors:** Bahar Aksan, Daniela Mauceri

**Affiliations:** 1 Department of Neurobiology, Interdisciplinary Centre for Neurosciences (IZN), Heidelberg University, INF 366, 69120 Heidelberg, Germany; 2 Institute of Anatomy and Cell Biology, Dept. Molecular and Cellular Neuroscience, University of Marburg, Robert-Koch-Str. 8, 35032 Marburg, Germany

**Keywords:** VEGFA, VEGFB, VEGFC, VEGFD, PIGF, Neurovascular, Neuronal structure, Neural development, Neuroprotection, Synaptic activity

## Abstract

Neurons rely on the bloodstream for essential nutrients and oxygen, which is facilitated by an intricate coupling of the neuronal and vascular systems. Central to this neurovascular interaction is the vascular endothelial growth factor (VEGF) family, a group of secreted growth factors traditionally known for their roles in promoting endothelial cell proliferation, migration, and survival in the cardiovascular and lymphatic systems. However, emerging evidence shows that VEGFs also play indispensable roles in the nervous system, extending beyond their canonical angiogenic and lymphangiogenic functions. Over the past two decades, VEGFs have been found to exert direct effects on neurons, influencing key aspects of neuronal function independently of their actions on vascular cells. In particular, it has become increasingly evident that VEGFs also play crucial functions in the development, regulation, and maintenance of neuronal morphology. Understanding the roles of VEGFs in neuronal development is of high scientific and clinical interest because of the significance of precise neuronal morphology for neural connectivity and network function, as well as the association of morphological abnormalities with neurological and neurodegenerative disorders. This review begins with an overview of the VEGF family members, their structural characteristics, receptors, and established roles in vasculature. However, it then highlights and focuses on the exciting variety of neuronal functions of VEGFs, especially their crucial role in the development, regulation, and maintenance of neuronal morphology.

## Introduction

The vascular endothelial growth factor (VEGF) family, part of the VEGF/ platelet-derived growth factor (PDGF) superfamily, consists of secreted glycoproteins. These proteins regulate endothelial cell proliferation, migration, and survival, playing essential roles in cardiovascular and lymphatic system development and maintenance [1]. In mammals, the VEGF family includes VEGFA—commonly known simply as VEGF-, placental growth factor (PlGF), VEGFB, VEGFC, and VEGFD [2–8]. VEGFD and VEGFC are classified as a subfamily within the VEGF family due to their close structural similarity [8, 9]. Additionally, VEGF-like proteins have been identified in Orf viruses (VEGFE) [10–12] and snake venom (VEGFF) [13–15]. A thorough phylogenetic analysis of the VEGF family is already available [16]. This review will focus on mammalian VEGFs.

## Protein structure of the VEGF family

Alternative splicing, protein glycosylation, and proteolytic processing lead to a further diversity of VEGF isoforms with distinct biochemical properties and receptor interaction affinities [17–20]. Proteolytic processing serves as a critical mechanism in the VEGF family, particularly for VEGFC and VEGFD, regulating their maturation and receptor affinities [9, 19, 21]. Most VEGFs exist as monomers or anti-parallel homodimers and VEGFA, PIGF and VEGFB additionally form heterodimers [5, 21–24]. The VEGF homology domain (VHD) supports dimerization through a cystine knot motif, a characteristic of the VEGF/PDGF superfamily [25–27]. The cystine knot motif consists of eight or, in the case of VEGFD and VEGFC, nine regularly spaced cysteine residues which form intra- and intermolecular disulfide bonds that play a crucial role for structural stability and dimerization of VEGFs [8, 25–28]. However, the additional cysteine residue in VEGFC and VEGFD reduces dimer stability and homodimers of proteolytically processed mature VEGFD and VEGFC are predominantly non-covalently bound [9, 21, 28, 29].

## Expression and non-neuronal functions of the VEGF family

The VEGF family is integral to vascular and lymphatic systems. VEGFA, expressed early in development in embryonic and extra-embryonic structures, regulates developmental vasculogenesis and angiogenesis with heterozygous loss resulting in embryonic lethality [30–34]. VEGFA expression decreases in adulthood but persists in almost every tissue and cell types including endothelial cells, pericytes, smooth muscle cells, cardiac and skeletal myocytes, epithelial cells, neurons, astrocytes, Schwann cells and microglia [35–43]. In adulthood, VEGFA modulates angiogenesis and vascular permeability while contributing to various physiological processes. These include vascular functions like vessel maintenance and wound healing, as well as non-vascular processes such as bone formation, hematopoiesis, lymphangiogenesis, and multiple functions in the female reproductive system [36, 44–53].

PlGF is broadly expressed in various tissues, including the brain, with high expression in the placenta during embryonic development [39, 54, 55]. VEGFB is found in the brain, spinal cord, heart, and skeletal muscle and is controversial for its direct angiogenesis inducer role [37–39, 56–64]. It primarily serves as a factor for cell survival and may have cardioprotective and anti-angiogenic effects [62–65]. Unlike VEGFA, VEGFB and PlGF are redundant for normal development and VEGFB- or PlGF-deficient mice only develop mild phenotypes [66, 67]. VEGFC and VEGFD also participate in angiogenesis, but their primary function is the regulation of lymphangiogenesis [68–70]. VEGFC, expressed during embryogenesis in lymph sacs and vessel sprouting regions, is critical for lymphatic system development with its absence being embryonically lethal [71, 72]. Its expression in adulthood, including heart, placenta, muscle, ovary, and small intestine, supports lymphatic maintenance and tissue repair [6, 39]. VEGFD, broadly expressed both during development and adulthood across tissues, including the heart, lungs, liver, skeletal muscle, colon, small intestine, skin, and brain, enhances lymphangiogenesis and angiogenesis but is nonessential for development while retaining the capacity to induce lymphatics growth when overexpressed [8, 21, 39, 73–80]. Notably, VEGFC and VEGFD exhibit distinct expression profiles in the brain; VEGFC expression is stronger during development in neural stem cells and decreases in the postnatal period where it is then primarily expressed by reactive astrocytes and microglia [81–84]. On the contrary, VEGFD is expressed throughout development and adulthood especially in hippocampus and cortex [75–78].

## VEGF family signaling

**Fig. 1:**
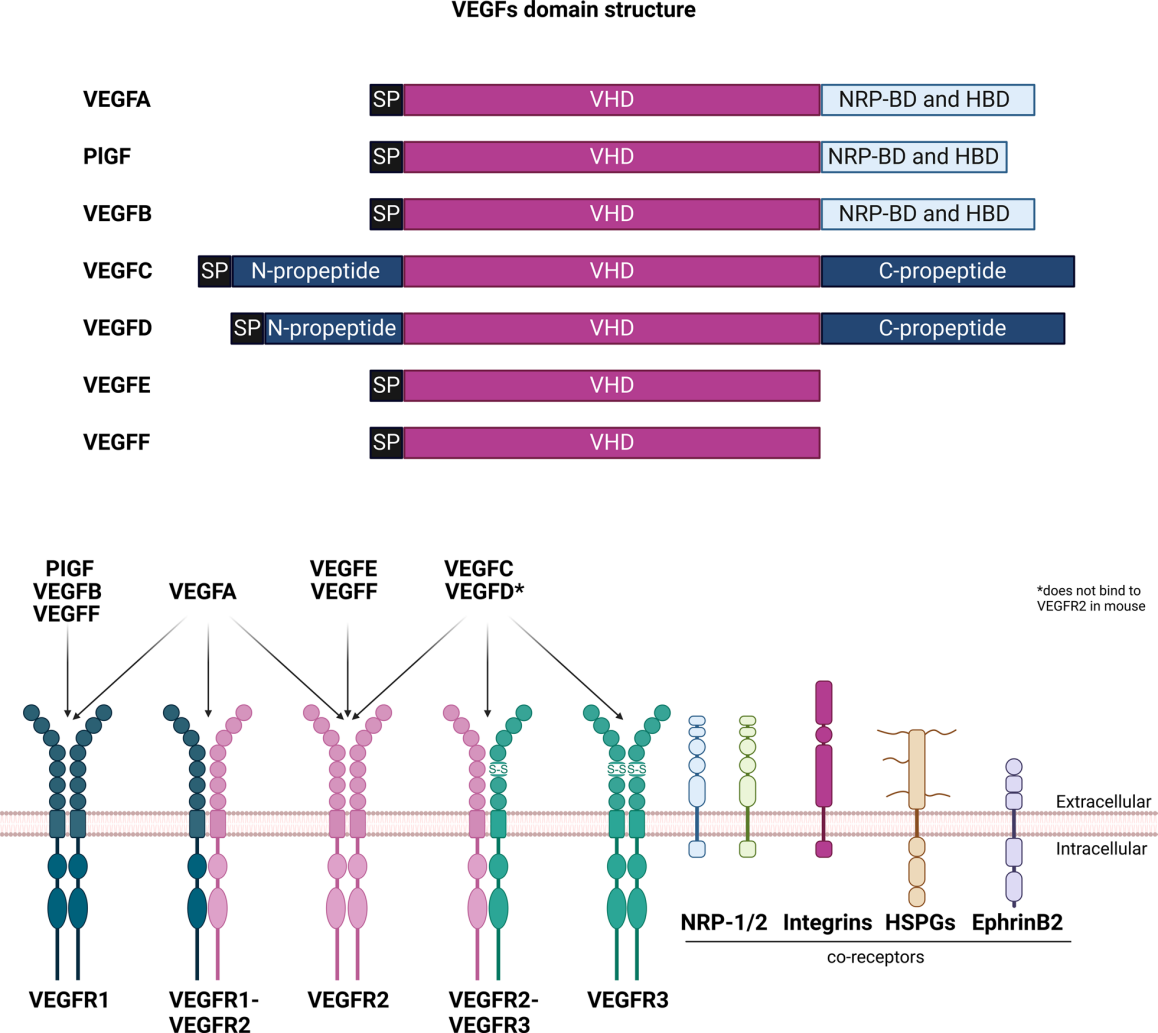
Structure and receptor specificity of VEGFs. Schema illustrating domain structures and binding affinities of VEGFs to their cognate VEGF receptors. The VEGF homology domain (VHD), which contains the cystine knot motif and VEGFR-binding sites, is highly conserved across VEGFs. The C-terminal domains harbor neuropilin- and heparin-binding domains (NRP-BD and HBD). The N- and C-terminal propeptides of VEGFC and VEGFD are proteolytically removed during protein maturation. The signal peptide (SP) controls protein secretion. Alternative splicing at domains flanking the VHD can lead to additional isoforms with different properties and binding affinities. For a detailed overview of splice isoforms, see the following excellent reviews [266, 267]. Beyond primary receptor interactions, VEGFs and VEGFRs engage with co-receptors neuropilin-1 (NRP-1), neuropilin-2 (NRP-2), and heparan sulfate proteoglycans (HSPGs), which modulate downstream signaling. VEGFRs also associate with integrins and EphrinB2, further influencing VEGF-mediated signaling pathways. Created in BioRender. https://BioRender.com/01r617

Although intracellular functions of VEGFs have been reported [85, 86], VEGFs primarily signal, after being secreted, by interacting with their cognate VEGF receptors (VEGFRs) (Fig. 1). VEGFRs are receptor tyrosine kinases (RTKs), and three types are known: VEGFR1 [also referred to as fms-like tyrosine kinase 1 (flt1)], VEGFR2 [also referred to as kinase insert domain-containing receptor (kdr), or fetal liver kinase 1 (flk1)] and VEGFR3 [also referred to as fms-like tyrosine kinase 4 (flt4)]. In addition to VEGFRs, secreted VEGFs can also interact with extracellular matrix (ECM) components, neuropilin-1 (NRP-1) and neuropilin-2 (NRP-2) co-receptors, and heparan sulfate proteoglycans (HSPG) [85–88]. They can either bind alone or in combination with VEGFR to modulate downstream signaling [87, 88] (Fig. 1). HSPGs and the ECM also control VEGFs availability or present VEGFs to their appropriate receptor, which affects VEGF-VEGFR binding and the subsequent downstream signaling cascades [87, 88]. For instance, HSPGs expressed on neighboring cells can present VEGFA to VEGFR2 [89].

The particular VEGF subtype, splice variant, and degree of proteolytic processing all affect how well VEGFs bind to their receptors, co-receptors, and ECM [1, 18, 19, 21, 27, 90–93]. The same VEGFR can bind to more than one VEGF. VEGFA interacts with VEGFR1, VEGFR2, and VEGFR1-VEGFR2 heterodimers while VEGFB and PlGF bind to VEGFR1; VEGFE binds to VEGFR2; and VEGFF interacts with both VEGFR1 and VEGFR2. The mature, proteolytically cleaved forms of VEGFC and VEGFD have a higher affinity for VEGFR3 but can also engage VEGFR2 or VEGFR2-VEGFR3 heterodimers [19, 21, 94, 95] (Fig. 1). Interestingly, species-specific binding differences, such as VEGFD’s exclusive VEGFR3 interaction in mice, underscore the complexity of VEGFs signaling [96]. The species-specific interaction derives from differences in VEGFD amino acid sequence at the binding surface rather than from differences in VEGFR2 [26].

Ligand binding induces the dimerization of VEGFRs to homo- or heterodimers, followed by activation of the receptors through autophosphorylation [87, 97]. Once activated, the tyrosine kinase domains trans-phosphorylate specific tyrosine residues on the cytoplasmic regions of the opposing receptor in the dimer. This trans-phosphorylation not only regulates the kinase activity but also modulates the receptor’s ability to interact with downstream signaling molecules, effectively linking receptor activation to intracellular signaling cascades [87, 94, 97]. The specific VEGFR subtype and ligand determine tyrosine phosphorylation patterns. These patterns are modified by interactions with accessory proteins, co-receptors, and ECM, leading to activation of distinct signaling pathways [87, 88, 98, 99]. In conclusion, VEGFs signaling’s complexity, driven by receptor-ligand and species-specific interactions with divergence of downstream pathways, underscores the future need for further detailed understanding of VEGFs biology in different cells and tissues.

## VEGF family in disease

As key regulators of the cardiovascular and lymphatic systems, altered VEGFs signaling is linked to various disorders, particularly tumor pathogenesis. Pathological aspects of deregulated VEGFs signaling have been extensively reviewed elsewhere and we invite the reader to a few excellent examples [32, 62, 63, 100–104]. In brief, VEGFA, central to vascular processes, is targeted in cancer and eye diseases through antibodies, decoy receptors, or inhibitors [32, 102, 105, 106]. Viral-based gene therapies delivering anti-VEGFA antibodies or soluble receptor forms are in clinical trials for ocular diseases [107, 108]. VEGFB’s cardioprotective properties make it promising for heart disease therapies, while PlGF inhibition shows potential in pathological angiogenesis [63, 104, 109]. VEGFC and VEGFD contribute to tumor angiogenesis, lymphangiogenesis, metastasis and ocular disorders, with therapeutic strategies under development [100, 101, 103]. Abnormal VEGFD levels serve as diagnostic markers in lymphangioleiomyomatosis and are associated with pulmonary conditions [100, 110]. VEGFD delivery strategies are being explored for lymphatic and vascular diseases [100]; including gene therapy for refractory angina in clinical trials [111]. While VEGFs show therapeutic promise, a deeper understanding of their specific roles in disease is crucial for developing precise medical treatments. Several knowledge gaps persist, particularly regarding off-target effects and how VEGFs functions vary across different cells, tissues, and disease states. A prime example is VEGFC, which influences both blood and lymphatic vessel formation, making its therapeutic application complex—beneficial for tissue repair but potentially problematic in cancer treatment. Moreover, as will be discussed in the remaining sections of this review, VEGFs also play multiple roles in neural cell function, adding another layer of complexity to their potential therapeutic use. To maximize their medical potential, researchers must focus on mapping out VEGFs signaling networks and their effects in specific biological contexts, both healthy and diseased.

## Relevance of VEGFs in neuronal contexts

While the impacts of VEGFs on the vascular system are extensive and thoroughly documented, VEGFs also play a role in the nervous system, which is also reflected by the neuronal expression of their receptors. VEGFR2 is widely expressed across diverse neuronal populations throughout development and adulthood, with particularly well-documented expression in the subventricular zone (SVZ), hippocampal CA3, cerebellum, cortex and dorsal root ganglia (DRG) [112–126]. Importantly, VEGFR2 localizes to sites essential for neuronal function, including axonal branching points, axonal growth cones, dendrites, and postsynaptic densities [116, 125–128]. Beyond neurons, VEGFR2 is also expressed in astrocytes [127]. The VEGFR2 co-receptor, NRP-1, shares a partially overlapping expression pattern, with specific localization to axons and growth cones [116, 122, 125, 127, 129–132]. In contrast, VEGFR1 is predominantly expressed in astrocytes of the adult mouse brain, whereas its expression is generally absent or low in developing neurons of the SVZ, hippocampus, cortex, retina and in the peripheral nervous system (PNS), with expression further declining in adulthood [114, 117, 118, 120, 125, 130, 132–135]. However, VEGFR1 expression can be induced by VEGFA [118]. VEGFR3, in turn, is expressed in neural progenitors and stem cells in the SVZ and subgranular zone (SGZ) throughout development and adulthood. It is also present in postmitotic neurons and astrocytes across multiple brain regions, including the hippocampus, cortex, and cerebellum, from early development into adulthood [81, 124, 136–140].

This review will next critically examine the nuanced roles of the VEGF family in neuronal contexts, including development, neuroprotection, and synaptic functions. Significant focus is directed towards the modulation of neuronal structure – including axons, dendrites, and spines – a subject that sparked increasing interest yet has not been comprehensively reviewed.

## Neurogenesis and neuronal migration: role of VEGFs

**Fig. 2:**
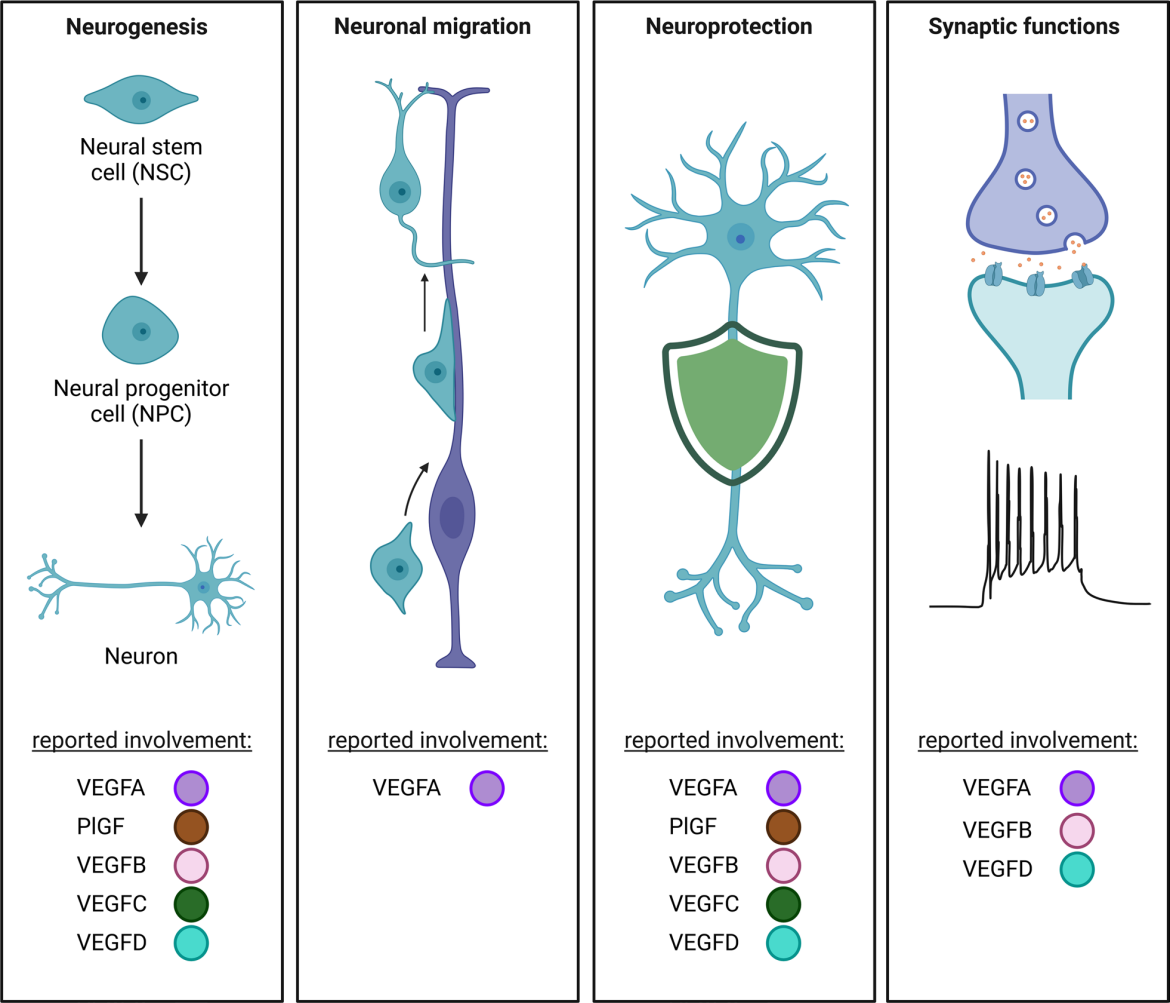
VEGFs regulate a broad range of neuronal functions. VEGFs play critical roles in neurogenesis, neuronal migration, neuroprotection, and synaptic function. This schema highlights the specific VEGFs involved in each neuronal function. Note that the assignment of a VEGF to a particular role in the schema does not indicate a strictly promotive role; VEGFs may also have inhibitory effects, which are not distinguished here for simplicity. Refer to the main text for detailed descriptions of VEGF functions. Created in BioRender. https://BioRender.com/w52v006

Neurons obtain essential nutrients and oxygen from the bloodstream, a process made possible by the intricate connection between nerves and blood vessels. This close relationship allows these systems to mutually influence each other's growth [141]. VEGFs lie at the core of this bidirectional neurovascular communication as, for instance, newly formed vessels, induced by VEGFA, release growth factors to regulate neurons, which in turn secrete VEGFA to guide blood vessel growth [141–143]. This interdependence phenomenon between neuronal and vascular components is particularly evident during neurogenesis, the formation of new neurons from neuronal stem cells (NSC; Fig. 2, Table 1) [144]. Despite ongoing debate regarding its role in the human brain, neurogenesis continues to take place in the rodent adult brain in the SVZ and the SGZ of the hippocampal dentate gyrus, where NSCs and blood vessels are closely associated and grow in a coordinated manner [145–150].

**Table 1:** Overview of the effects of VEGFs in neurons

	VEGFA	PlGF	VEGFB	VEGFC	VEGFD
Neurogenesis	↑ VEGFR2 [123, 154–161]	↓ VEGFR1 [154]	↑ VEGFR1 [123, 165, 167]	↑ VEGFR3 [81, 137, 138, 168]	↑ [169, 170]
Neuronal migration	↑ VEGFR2, NRP-1 [112, 120, 121, 129, 173, 174]				
Neuroprotection	↑ VEGFR2, NRP-1 [41, 122, 131, 167, 175–194] no effect [139]	↑ [195–197] no effect [122] ↓ [198]	↑ VEGFR1 [60, 132, 134, 135, 199–207]	↑ [208] no effect [139]	↑ [139, 211]
Synaptic functions	↑ VEGFR2 [114, 119, 128, 194, 213–216, 219, 221, 222, 224] ↓ [217, 218, 223]		↑ [227]		↑ [76, 225, 226]
Axon morphology					
Growth cone organization	↑ VEGFR2, NRP-1 [116, 130]	↑ NRP-1 [130]			no effect [130]
Growth cone guidance and turning	↑ VEGFR2, NRP-1 [113, 234]			no effect [113]	
Neurite outgrowth	↑ VEGFR2, NRP-1 [115, 117, 118, 231–234] no effect [113]	no effect [117]	↑ VEGFR1 [199] no effect [117]		
Axon or neurite elongation	↑ VEGFR2 [125, 132]	↑ NRP-1 [132, 236]	↑ VEGFR1, NRP-1 [132]	no effect [125]	↑ [125]
Axon or neurite branching	↑ VEGFR2 [125, 132]		↑ VEGFR1, NRP-1 [132]	no effect [125]	no effect [125]
Dendrites and synapses					
Developmental dendritogenesis	↑ VEGFR2 [118, 126, 237–239] no effect [238]				
Adult dendrite remodeling	no effect[237, 239]				↓ VEGFR3 [124] ↑ VEGFR3 (PNS) [242]
Dendrite elongation					↓ VEGFR3 [124]
Dendrite branching	↑ VEGFR2 [126, 237]			↑ VEGFR3 [242]	↓ VEGFR3 [124] ↑ VEGFR3 (PNS) [242]
Dendrite maintenance	no effect [76, 124, 139, 211]			no effect [76, 124, 139, 211, 257]	↑ VEGFR3 [76, 124, 139, 211, 225, 226, 242, 251, 257]
Spinogenesis (developmental)	↑ VEGFR2 [126, 215, 237]				
Spinogenesis (adult)	↑ [240, 241] ↓ [237]				no effect [76, 124, 226]

Upward arrows (↑) represent a VEGF family-induced upregulation or requirement for the process. Downward arrows (↓) indicate downregulation or suppression

Several studies have shown that VEGFA plays a crucial role in adult neurogenesis, which have been extensively reviewed elsewhere [43, 151–153]. In brief, VEGFA is essential for neurogenesis in adult rodents induced by exercise, learning, anti-depressants, electroconvulsive seizures, and environmental enrichment. It can also enhance recovery following brain injury or ischemia by promoting neurogenesis [154–161]. Both chronic stress and aging reduce VEGFA expression, which correlates with decreased neurogenesis [162, 163]. However, the molecular mechanisms underlying its effects remain incompletely understood, particularly in distinguishing its vascular-dependent and independent roles via a direct effect on VEGFR2-expressing neuronal precursors [123, 164].

VEGFB also promotes neurogenesis but exhibits distinct mechanisms [165]. VEGFB's effects are mediated through VEGFR1 and NRP-1, with a lesser extent of neurogenesis in vivo compared to VEGFA, raising questions about the differential roles of VEGFR1 and VEGFR2 activation in neurogenic processes [5, 123, 165–167]. Interestingly, activation of VEGFR1 through PlGF reduces hippocampal neurogenesis and VEGFA-VEGFR1 signaling in glia cells somehow exerts a negative effect on adult olfactory neurogenesis in mice [133, 154].

VEGFC also can promote both developmental and adult neurogenesis. VEGFC, differently than VEGFA, VEGFB and PIGF, primarily exerts its neurogenic properties via VEGFR3 in what appears to be a conserved mechanism. Downstream of VEGFR3, VEGFC activates extracellular signal-regulated protein kinase (ERK) and Akt pathways, driving cell proliferation in human embryonic stem cell models [81, 137, 138]. Several studies indicate that VEGFC-induced neurogenesis is a direct effect on neurons rather than vessel-mediated, as VEGFC did not induce angiogenesis in the brain, and conditional deletion or inactivation of VEGFR3 in NSCs interfered with neurogenesis [81, 137, 138]. Notably, VEGFR3 knockout-mediated disruption of neurogenesis correlated with heightened fear responses. This finding emphasizes the role of VEGFC-VEGFR3 signaling in behavior-associated neurogenesis and indicates its potential as a therapeutic agent for psychiatric and neurodegenerative disorders [138]. Indeed, injecting microbeads that contain NSCs in combination with VEGFC-secreting endothelial cells into the mouse brain promoted NSC proliferation and was beneficial against hemorrhagic stroke in the mouse brain [168].

VEGFD, though less studied, has also been associated to the induction of adult hippocampal neurogenesis and improvements in spatial learning and memory in mice [169]. Moreover, VEGFD also induces the differentiation of human embryonic stem cells into dopaminergic neurons in vitro, positioning it as a candidate for further exploration in regenerative therapies [170].

During development but also in adulthood, new-born neuroblasts migrate from their birthplace to their intended destination [171]. Much like its role in directing the movement of endothelial cells, VEGFA has been identified as a chemoattractant that guides neuronal migration through distinct molecular mechanisms across developmental stages and brain regions (Fig. 2, Table 1)[1, 43, 151–153, 172, 173]. During embryonic development, VEGFA interacts with NRP-1 to guide cranial neural crest migration from the neural tube to specific branchial arches in the chick embryo and the facial branchiomotor neurons in the developing mouse hindbrain [112, 129, 174]. In postnatal development, VEGFA guides migration through neuronal VEGFR2-dependent mechanisms. For example, in the cerebellum, VEGFA-activated VEGFR2 forms complexes with N-methyl-D-aspartate receptor (NMDAR) subunits, activating Src family kinases that phosphorylate the NMDAR subunit GluN2B, ultimately enhancing NMDAR-mediated currents and Ca^2+^ influx [121]. In rat SVZ cultures and explants, VEGFA directs the migration of Fibroblast Growth Factor 2 (FGF-2)-stimulated neuronal progenitor cells also through neuronal VEGFR2 [120]. Both VEGFR2-dependent processes require specific ECM-bound VEGFA splice isoforms—VEGF164 and VEGF188—to generate essential concentration gradients. Soluble VEGF120, in contrast, fails to accomplish this [120, 121]. Meanwhile, glia-expressed VEGFR1—which also had negative effects on neurogenesis itself—acts as a counterbalance, restraining neuronal progenitor migration along the rostral migratory stream [133]. Despite important advances, unresolved questions remain regarding the spatiotemporal dynamics of VEGFs in neurogenesis and neuronal migration. Understanding how the different VEGFs signaling elements integrate with other molecular pathways previously linked to such phenomena [i.e. Wnt, Notch, Epidermal growth factor (EGF)] could provide deeper insights. Moreover, valuable input may derive from an expansion of experimental work addressing the functional consequences of VEGFs-regulated neurogenesis on different behavioral aspects such as anxiety, fear, or learning. Importantly, the currently available information is largely based on animal models and future work should aim at defining the role of VEGFs in human neurogenesis as this will be critical to eventual translation of findings into clinical contexts.

## VEGFs in synaptic functions

In the last years, it has also become clear that VEGFs modulate neuronal synaptic functions both at the pre- and post-synaptic side (Fig. 2, Table 1).

For instance, VEGFA plays a critical role in synaptic excitability, transmission, and plasticity [152, 153, 213, 214]. VEGFA, independent of neurogenesis or angiogenesis, enhances synaptic strength and plasticity through VEGFR2 signaling and blocking VEGFA reduces certain properties of action potentials in CA1 pyramidal neurons [128, 215]. More precisely, VEGFA enhances NMDAR-mediated excitatory postsynaptic currents (EPSCs) and the frequency of miniature EPSCs (mEPSCs), indicative of increased presynaptic neurotransmitter release, in hippocampal cultures and slices [128]. VEGFA also promotes long-term potentiation (LTP) thereby regulating hippocampal-dependent memory [114, 128, 215, 216]. These findings underscore VEGFA’s role in synaptic plasticity and memory regulation. Paradoxically, in certain contexts, VEGFA also exhibits inhibitory effects on synaptic transmission. It reduces amplitude and frequency of field excitatory and inhibitory postsynaptic potentials (EPSPs and IPSPs) in hippocampal and motor neurons but does not alter motor neuron firing properties in brainstem slices [217, 218]. Conversely, in spinal motor neurons, VEGFA induces phrenic motor facilitation, enhancing nerve burst frequency and amplitude [219]. These contradictory findings may reflect differences in the used experimental models, neuronal cell types, VEGFA dosages, or interactions with other signaling pathways, such as Notch1 [220].

The mechanisms underlying VEGFA’s influence on synaptic functions remain only partially understood but have been shown to involve the modulation of ion channels, including voltage-gated Na^+^ channels (L-VACC), delayed-rectifying K^+^ channels, and high-voltage-activated Ca^2+^ channels [114, 194, 221, 222]. Through VEGFR2, VEGFA increases intracellular Ca^2+^ levels, likely via Ca^2+^ influx through L-VACCs, NMDARs, and voltage-independent transient receptor potential canonical channels, and release from internal stores through PLCγ-IP3 (phospholipase Cγ-inositol 1,4,5-triphosphate) pathway activation [114]. For instance, VEGFA enhances NMDAR-mediated currents by facilitating the interaction between VEGFR2 and the GluN2B subunit of NMDARs in cerebellar granule cells, which triggers SFK-dependent GluN2B phosphorylation and increases Ca^2+^ influx [119]. Additionally, the co-application of VEGFA and NMDA promotes the recruitment of GluN2B to postsynaptic sites, thereby enhancing NMDAR-induced currents and driving coordinated remodeling of both NMDARs and α-amino-3-hydroxy-5-methyl-4-isoxazolepropionic acid receptors (AMPARs) in hippocampal neurons [128]. This remodeling activates downstream signaling pathways, such as calcium–calmodulin-activated kinase (CaMKII) and protein kinase C (PKC) signaling [128]. Furthermore, VEGFA upregulates the expression of AMPAR subunit GluR2, which reduces the Ca^2+^ permeability of AMPARs [223]. VEGFA-induced synaptic plasticity may also involve structural changes, including alterations in spine and dendrite density and morphology [128, 214, 215].

Interestingly, VEGFA expression is activity-dependent, linking it to synaptic activity both as a regulator and a consequence. Membrane depolarization, Ca^2+^ influx, and seizure-induced activity increase VEGFA levels, a process that depends on the activation of NMDAR or L-type voltage-gated channels [114, 179, 217]. Additionally, Hypoxia-Inducible Factor-1α also stimulates VEGFA expression, thereby enhancing excitatory synaptic transmission [224]. Elevated VEGFA levels may not only play a crucial role in modulating synaptic functions but also act as a feedback regulatory mechanism during periods of heightened activity. For instance, the upregulation of VEGFA following seizures may function to limit excitability [217].

VEGFD is crucial for maintaining dendrite morphology and, consequently, for supporting network activity. Loss of VEGFD results in the simplification of basal dendrites in pyramidal cells in vitro and in vivo and impairs the formation of hippocampus-dependent spatial and contextual fear memories [76, 225, 226]. The reduction in dendritic complexity and, consequently, in plasma membrane surface area leads to impaired activity-dependent nuclear calcium signaling and diminished network activity as measured by reduced spike frequencies, neuronal excitability, and membrane capacitance in vitro [76, 225]. Resting or threshold membrane potentials are, however, not affected [76]. Additionally, measurements of mEPSCs and responses to bath-applied AMPA indicate fewer AMPAR-containing synapses, fewer AMPARs within those synapses and a total reduction of AMPARs [76]. Surprisingly, VEGFD knockdown in vivo, despite causing memory deficits, does not alter intrinsic electrical properties of hippocampal CA1 neurons as membrane capacitance, action potentials, and accommodation were unaffected [225, 226]. Likewise, no changes are detected in local field potentials, cross-frequency phase-amplitude coupling of theta and gamma oscillations and sharp-wave ripples [226]. This discrepancy may be attributed to VEGFD’s region-specific effects, as VEGFD knockdown reduces the length and complexity of basal dendrites, while apical dendrites exhibit increased length and complexity [226]. VEGFD further prevents synaptic activity-induced dendritic remodeling without impairing neuronal activity [124].

While VEGFA and VEGFD emerged as crucial for synaptic function, little is known about the role other VEGF family members. Nevertheless, VEGFB was shown to restore alterations in firing and synaptic input caused by axotomy in motor neurons [227]. Moreover, a recent study suggests a potential role for VEGFC in inhibitory and interneuron activity, as its overexpression upregulated the transcription of calcium signaling regulatory genes in these neurons [209].

In summary, VEGFs are increasingly recognized as key modulators of synaptic functions. VEGFA, the most extensively studied, has complex dual roles in both enhancing synaptic plasticity and excitability while inhibiting them in certain contexts, underscoring the need for further research to harness its therapeutic potential. VEGFD is crucial for maintaining dendritic morphology and network activity, though its region-specific effects highlight the importance of understanding its mechanisms and sites of action. Research on other VEGF family members remains limited, but their potential therapeutic applications in neurology demand further investigations to fully explore their benefits and expand treatment options.

## Functions of VEGFs in neuronal morphology

**Fig. 3:**
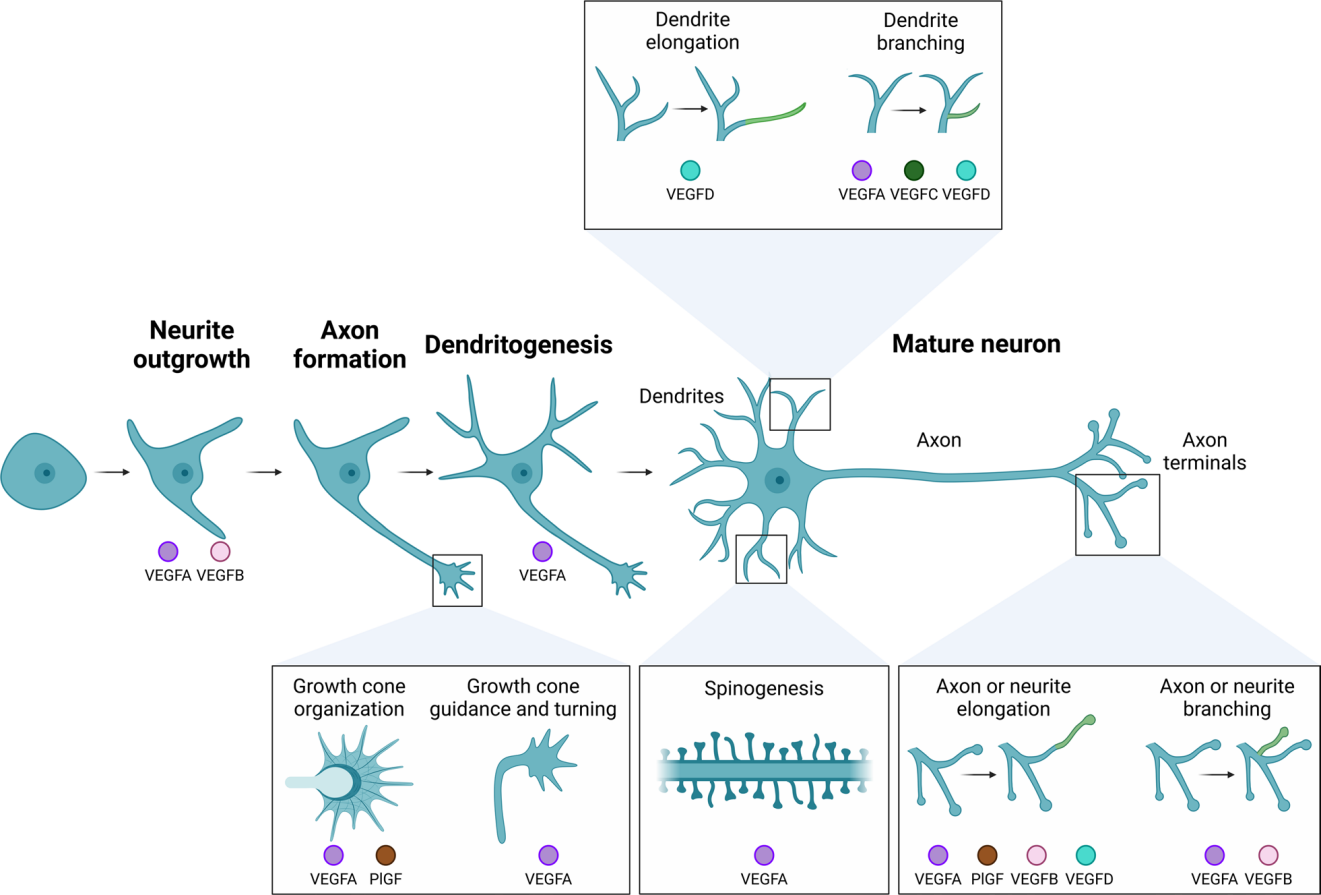
Role of VEGFs in neuronal morphology. VEGFs regulate numerous aspects of neuronal morphology, including neurite outgrowth, growth cone organization, axonal guidance and turning, dendritogenesis, elongation and branching of neurites, axons, and dendrites, as well as spinogenesis. Through these mechanisms, VEGFs contribute to the development, regulation, and maintenance of neuronal structure. This schema shows the specific VEGFs involved in different morphological compartments of neurons. Note that the display of a VEGF in a particular compartment in the schema does not indicate a strictly promotive role; VEGFs may also have inhibitory effects, which are not distinguished here for simplicity. Refer to the main text for detailed descriptions of VEGF functions. Created in BioRender. https://BioRender.com/o56d588

The polarized shape of neurons enables signal transmission and reception. A single long projection, the axon, transmits impulses to other neurons, while shorter, highly branched dendrites receive and process incoming signals [228]. Synaptic communication occurs at the junction between axons and dendrites, with excitatory synapses typically located on small protrusions from dendrites known as dendritic spines [229]. Traditionally viewed through a vascular lens, VEGFs now emerge as essential modulators of neuronal architecture affecting axons, dendrites, and spines (Fig. 3, Table 1).

## Axon growth and guidance

VEGFs play pivotal roles in axonal guidance and growth, with VEGFA as the most extensively studied member of the family in this context. Axonal growth cones, a dynamic structure at the tip of developing axons, share striking structural and functional similarities with endothelial tip cells at the leading edge of vascular sprouts [230]. Similar to its role in guiding endothelial tip cells, VEGFA promotes axonal growth, guidance, and the outgrowth of neuronal processes, also known as neurites, in a variety of neuronal systems [153, 172]. For instance, VEGFA promotes neurite outgrowth in axotomized postnatal retinal RGCs, embryonic glial-reduced ventral mesencephalic neuronal cultures, and explants of adult mouse superior cervical ganglia (SCG) or DRG [231–233]. While the mitogenic effects of VEGFA on Schwann cells may indirectly support neurite outgrowth in SCG and DRG models [233], compelling evidence suggests that VEGFA acts directly on neurons. Through VEGFR2, VEGFA stimulates neurite outgrowth in rat and mouse primary cortical neurons, independent of astroglial or angiogenic influences [115, 117, 118]. Key signaling pathways such as Rho/ Rho-associated protein kinase (ROK) signaling, phosphorylation of the actin-regulatory protein cofilin and the MAPK and PI3K/Akt signaling pathways mediate this effect [115, 117, 118]. Moreover, VEGFA reorganizes actin cytoskeleton within the growth cones, increasing their area and motility via VEGFR2 and NRP-1 in chicken DRG neurons [116]. Interestingly, VEGFR2 interacts with the NRP-1-PlexinD1 complex and is activated by Semaphorin 3E in embryonic mouse subicular neurons, promoting axon elongation independently of VEGFA [127].

VEGFA’s role in axonal structure extends beyond promoting outgrowth as it also plays a role in axonal guidance. In the developing mouse spinal cord, VEGFA secreted by the floor plate acts as a chemoattractant for commissural axons without influencing axonal outgrowth [113]. Disruption of VEGFA expression or VEGFR2 activity impairs axon guidance and turning, mediated by local activation of SFKs at the growth cones of commissural neurons [113]. Similarly, VEGFA influences optic chiasm crossing of commissural RGC axons through NRP-1 signaling [234]. Notably, VEGFA prevents growth cone collapse in developing rat DRG explants through NRP-1, even in the absence of *VEGFR1-* and *2* [130]. These observations highlight distinct receptor-ligand interactions underpinning VEGFA’s diverse roles in axon development. VEGFA-VEGFR2 also promotes elongation of axons and axonal branching, a critical process where axons extend filopodia that mature into new branches [235]. In cultured mouse hippocampal neurons, VEGFA stimulates axon branching by triggering internalization of VEGFR2, activating SFKs, and localizing to actin-rich locations along the axon that function as branching points [125]. This process occurs independently of the co-receptors NRP-1 and EphrinB2. In contrast, dendritogenesis requires EphrinB2 for VEGFR2 internalization and VEGFA-induced dendritic branching [125, 126]. VEGFR2’s essential role in stabilizing newly formed axon branches is evident, as its knockdown in the CA3 region of the mouse hippocampus, increases filopodia formation but results in immature, shorter axons that fail to form functional synapses with CA1 neurons [125].

In summary, VEGFA directly promotes axonal growth cone organization, axon growth, guidance and branching through diverse mechanisms that vary across neuronal systems depending on cell-type and context. While VEGFR2-mediated signaling predominates, regulating cytoskeletal dynamics and growth cone motility, other receptors, and co-receptors including NRP-1 and PlexinD1, and their interactions with additional ligands like Semaphorin 3E play critical roles. However, significant gaps still remain in understanding how VEGFA integrates with other guidance cues to shape neuronal networks. Further research should focus on defining the exact receptor-ligand interactions and downstream signaling pathways in diverse neuronal contexts in order to fully elucidate the therapeutic potential of VEGFA in neural regeneration and repair.

Other VEGF family members also exhibit axonal effects, albeit less extensively studied than VEGFA and in specific scenarios. For example, VEGFB or PlGF do not induce neurite outgrowth in mouse cortical neurons, where their receptor VEGFR1 is not expressed [117]. On the other hand, a specific PlGF isoform (PlGF2) that binds to NRP-1, but not the VEGFR1-specific PlGF1 isoform, prevents growth cone collapse of developing rat DRGs [130]. And induces neurite elongation in mouse trigeminal ganglia neurons [132]. Moreover, PlGF stimulates DRG neurite length extension in co-cultures of pancreatic ductal adenocarcinoma cell line and primary mouse DRGs. This, in turn, aids the invasion of cancer cells into nerves [236]. VEGFB seems more effective at increasing neurite length than PlGF2. VEGFB stimulates neurite outgrowth in primary neurons from the rat cerebellum, hippocampus and retina [199]. Neutralizing VEGFR1 abrogates this effect, indicating its critical role in mediating VEGFB-driven neurite growth [199]. Additionally, VEGFB promotes neurite elongation and branching in trigeminal ganglia neurons in the PNS more robustly than VEGFA and independently of vascular influences [132]. VEGFB-induced neurite growth is mediated through VEGFR1, NRP-1, and the PI3K-Akt and Notch signaling pathways [132]. In a mouse corneal injury model where the subbasal corneal epithelial plexus is removed to injure superficial trigeminal nerve endings, VEGFB plays a critical role in nerve regeneration and functional recovery [132]. Notably, the study showed that VEGFB-induced nerve regeneration was highly specific, targeting only the injured nerves leaving uninjured nerves unaffected. When VEGFB was administered, its effects were more localized in cases of small, localized injuries compared to larger injury areas [132]. Given the avascular nature of this model, indirect effects via the vasculature are fully excluded [132].

VEGFC and VEGFD, by contrast, exhibit limited roles on axon structure. VEGFC does not affect turning of commissural axons nor induces axon elongation and branching in hippocampal neurons [113, 125]. Distinctly different than VEGFA, VEGFD modestly increases axon length in developing hippocampal neurons, but does not promote branching or affect growth cone formation of developing DRGs [125, 130].

In conclusion, the currently available evidence positions VEGFA as the primary regulator of axonal development, with VEGFB and PlGF contributing under certain conditions while VEGFC and VEGFD exhibit minimal influence (Fig. 3, Table 1). However, the findings also emphasize the need for further investigation into the less-explored VEGF family members. Such research could uncover novel roles for these factors in axonal development and highlight their therapeutic potential in neural regeneration and repair.

## Dendrites and synapses

VEGFA has been widely studied for its involvement in dendrite and spine morphology [153, 172]. It promotes dendritic arborization and spine morphogenesis, particularly during developmental stages. For instance, in cultured rat cortical neurons, VEGFA promotes the developmental outgrowth of neurites and their maturation into dendrites through neuronal VEGFR2 and MAPK signaling [118]. On the other hand, sequestering VEGFA in adult-born neurons, such as those in the mouse olfactory bulb, leads to reduction in dendrite arborization and spine density without affecting the vasculature or perfusion [237]. VEGFA inhibition through bevacizumab (an anti-VEGFA antibody) also reduces spine number and length and impairs synaptic plasticity [215]. Intriguingly, while VEGFA supplementation increases total dendrite length in developing hippocampal and cortical neurons, VEGFA inhibition through bevacizumab also initially increases total dendrite length but later reduces it in hippocampal neurons, while no such reduction occurs in cortical neurons, indicating time- and cell type-dependent effects [238]. In neonatal or early developmental stages, when neurons have few stem dendrites and dendritic spines, VEGFA supplementation enhances dendritogenesis. In mature neurons, VEGFA’s effects are less straightforward. Indeed, mature neurons, which already possess complex dendritic structures, do not show significant changes in dendrite arborization following VEGFA treatment [76, 239]. Recently, it was shown that VEGFA promotes dendritic arborization, spine maturation, and synaptic plasticity in neurons of the CA3 region of the developing mouse hippocampus. This occurs by clustering of VEGFR2 and EphrinB2 complexes at postsynaptic sites, followed by endocytosis, which leads to activation of SFKs and Akt signaling pathways [126]. In contrast, conditional neuron-specific knockout of VEGFR2 or EphrinB2, results in reduced dendrite arborization, decreased spine density, impaired synaptic plasticity and less mature spines, characterized by smaller head size and an increased proportion of immature filopodia [126]. Interestingly, blocking VEGFA does not affect established, mature dendrites, indicating that VEGFA is essential for dendritogenesis but not for dendrite maintenance [76, 237]. However, blocking VEGFA after neuronal maturation paradoxically promotes spine formation [237]. At the same time, environmental enrichment-induced increase in spine density in CA1 pyramidal neurons of the hippocampus relies on enhanced VEGFA expression and VEGFR2 signaling [240]. VEGFA also offers neuroprotective effects, protecting mouse hippocampal neurons against spine loss, spine morphological alterations, and synaptic dysfunction when exposed to synthetic amyloid-β oligomers [241]. In summary, VEGFA plays a crucial role in dendrite arborization, spine morphogenesis, and synaptic plasticity during neuronal development, but appears to be dispensable for the maintenance of mature dendritic structures (Fig. 3, Table 1). Its role in spines within mature neurons, however, remains ambiguous. While VEGFA may negatively regulate spine density under specific conditions, it enhances spine formation in response to environmental enrichment. Moreover, there are cell-type or brain-region specific differences in VEGFA’s regulation of dendrite structure. These contrasting findings highlight the need for further investigation into VEGFA's context-dependent effects to better elucidate its therapeutic potential.

The role of VEGFC in dendrite arborization and spine morphogenesis remains largely unexplored. However, it was shown that human induced pluripotent stem cells (hiPSCs), differentiated into NSCs and cultured long-term in the presence of a VEGFR3-specific mutant VEGFC, develop more complex dendritic trees while maintaining the same average dendrite length [242]. Moreover, chronic VEGFC exposure enables adaptive dendrite remodeling, conferring neuroprotection against neurotoxic stress both in developing and mature hiPSC-NSCs [242]. In contrast, acute VEGFC treatment does not induce similar effects, suggesting that VEGFC’s benefits may be time-dependent or require prolonged exposure to exert effects on dendrite structure and neuronal resilience [242]. These findings suggest that VEGFC may offer a promising strategy for promoting dendritic remodeling and enhancing neuronal survival under stress. However, the lack of acute effects implies that its therapeutic application would require more nuanced delivery strategies, particularly in diseases characterized by both developmental and neurotoxic challenges.

In contrast to VEGFA and VEGFC, multiple evidence suggests that VEGFD may be crucial primarily for the maintenance and stability of dendrites in mature neurons. Loss-of-function experiments indicate that knockdown of VEGFD leads to simplified dendritic arbors of mature hippocampal neurons both in vitro and in vivo [76, 225, 226]. A similar, simplified, dendritic phenotype was achieved when VEGFR3 expression was targeted supporting the notion that VEGFD signaling is essential for dendrite maintenance [76]. Indeed, reducing expression of VEGFA or VEGFC had no effect [76]. Interestingly, in vivo studies show that VEGFD’s effects are region-specific: while length and complexity of basal dendrites in the CA1 region were reduced, apical dendrites, which feature distinct branching patterns and receive inputs from different layers, exhibited increased length and complexity upon reduction of VEGFD expression [226]. This regional variability could suggest that VEGFD signaling functions differently in different dendritic domains, that VEGFR3 is differentially localized or that compensatory mechanisms are at play [226]. Impairments in hippocampus-dependent spatial and contextual fear memory formation result from the loss of VEGFD in the hippocampus and the subsequent simplification of dendritic architecture [76]. Memory deficits likely result from the loss of basal dendrites in CA1 pyramidal cells, which receive input from the CA2 region [226, 243]. This region also generates sharp wave-ripple complexes essential for long-term memory consolidation [244]. In several neurological conditions, dendritic structure preservation is frequently compromised [245–247]. In mouse models of stroke and retinal degeneration, VEGFD levels decrease significantly, leading to dendritic loss, while VEGFD supplementation preserves dendritic structure and neuronal function [139, 211]. Notably, this protective effect is specific to VEGFD and does not extend to VEGFA or VEGFC [139, 211]. The preservation of dendrites in response to VEGFD signaling was shown to be mediated through neuronal VEGFR3 expression [211]. Dysregulated expression of neuronal calcium buffers has been associated with Alzheimer’s disease (AD), schizophrenia, and aging [248–250]. Elevating nuclear calcium buffering capacity by expressing a nuclear targeted form of the calcium buffer protein parvalbumin in mature hippocampal neurons reduces VEGFD expression, leading to decreased dendrite complexity [251]. Notably, these deficits can be reversed through administration of recombinant VEGFD [251]. Similarly, the nuclear accumulation of Histone Deacetylase 4 (HDAC4), an epigenetic regulator implicated in several neurodegenerative disorders—including stroke, PD, AD, and ataxia telangiectasia—also disrupts dendrite architecture of mature neurons by negatively affecting VEGFD expression [252–257]. The loss of dendrites caused by nuclear HDAC4 can be minimized by overexpressing VEGFD or treating with recombinant VEGFD, but not its homolog VEGFC [257].

While preserving established connections in mature neurons is vital, dendritic structural plasticity is crucial to development and adult cognitive functions [258–261]. During dendritogenesis, VEGFD levels are low, suggesting that VEGFD’s role in maintaining dendritic structure may be unnecessary—or even counterproductive—during dendrite development [76]. In adult neurons, dendrite remodeling signals, such as synaptic activity or fear memory formation, downregulate VEGFD expression in the hippocampus both in vitro and in vivo [124]. However, unlike the abrupt loss of VEGFD brought on by toxic stimuli, activity-induced VEGFD downregulation happens gradually and in a controlled manner during remodeling. Interestingly, dendritic structural remodeling requires VEGFD downregulation, as overexpression or supplementation of VEGFD—by activating VEGFR3—prevents remodeling [124]. Other members of the VEGF family, such as VEGFA or VEGFC, do not influence activity-dependent structural plasticity. VEGFD appears to maintain dendritic structure in a homeostatic manner by preventing dendrite elongation and destabilizing newly formed dendrites [124]. Blocking VEGFD downregulation during fear memory-induced dendritic remodeling intriguingly enhances spatial memory, suggesting that VEGFD downregulation may act as a mechanism for limiting memory formation [124]. Memory suppression is increasingly recognized as a biological necessity to regulate and limit memory formation [262]. In the PNS, VEGFD-VEGFR3 signaling reorganizes dendritic structure in neurons that innervate subcutaneous adipose tissue and protects them against structural remodeling induced by neurotoxicity [242].

This dendritic maintenance partially relies on p38 MAPK signaling activation by VEGFD-VEGFR3 [76]. VEGFD signaling also activates ERK1/2 and cAMP response element-binding protein (CREB), but does not affect the phosphorylation of MSK1 (mitogen stress-activated kinase 1), ATF2 (Activating transcription factor 2), Akt, MKK4 (Mitogen-activated protein kinase kinase 4), JNK (c-Jun N-terminal kinase), p70, CaMKII, GSKα/β (Glycogen synthase kinase 3 α/β) [76]. Although SFKs play a role in VEGFA-VEGFR2 signaling for dendrite and axon development and in VEGFC-VEGFR3 signaling in lung adenocarcinoma cells, they are not involved in VEGFD-VEGFR3-mediated stabilization of mature dendrites [124–126, 263]. VEGFD controls dendritic morphology by influencing the cytoskeleton. It activates via dephosphorylation the striatal-enriched protein tyrosine phosphatase (STEP), possibly via the phosphatase calcineurin, which in turn controls the phosphorylation of the cytoskeleton-regulatory protein ezrin [124]. Additionally, VEGFD signaling slows microtubule dynamics in dendrites and enhances cell stiffness, further contributing to its role in maintaining dendritic stability [124, 264].VEGFD, however, does not influence spine density or shape [76, 226] nor does it modulate actin spine dynamics [124]. This suggests that VEGFD’s role is confined to dendritic structure, not spine plasticity.

To date, there is limited evidence regarding the roles of VEGFB and PIGF in dendrite and spine morphogenesis. While these factors are known to influence neurite extension (see previous section)—an early stage in the formation of dendrites and axons—it remains unclear whether they regulate dendritic structure directly. Given their potential role in neurite dynamics, further research is needed to investigate their effects on dendritic morphology and their possible involvement in dendritic development or maintenance.

The contrasting roles of VEGFA, VEGFC, and VEGFD in dendritic and spine dynamics create a complex picture of VEGFs signaling in the regulation of neuronal structure. While VEGFA plays a dominant role in dendritic growth and spine formation during early development, its influence on mature neurons is quite specific to different scenarios and may vary based on environmental factors, such as enrichment or synaptic activity. In contrast, VEGFD is primarily involved in maintaining dendrite integrity in mature neurons and providing protection to dendrites, and thus, neurons, against neurotoxic stress but does not regulate spine formation (Fig. 3, Table 1). Taken together, these divergent roles suggest that VEGFA, VEGFD, and VEGFC are thus likely to function in a complementary manner during different stages of dendrite development, with VEGFA and VEGFC promoting growth and remodeling, and VEGFD ensuring maturity and stability. Further studies are required to elucidate these complexities and may lead to a clearer picture.

## Neuroprotection and VEGFs: a therapeutic promise

The potential neuroprotective effects of VEGFs extend across diverse neurological conditions, presenting an exciting avenue for therapeutic development. Members of the VEGF family have demonstrated intriguing neuroprotective capabilities in several disease models, with both direct and indirect mechanisms implicated.

VEGFA is the most extensively studied member of the VEGF family in neuroprotection (Fig. 2, Table 1). VEGFA showed neuroprotective and neuroregenerative effects in animal models of epilepsy, cerebral ischemia, brain injury, peripheral nerve injury, and neurodegeneration [167, 172, 175–185]. While some of these effects can be attributed to VEGFA’s angiogenic and neurogenic properties, a growing body of evidence highlights direct neuroprotective mechanisms [153]. For instance, studies have shown that VEGFA directly protects cultured neurons from toxic stimuli, including excitotoxicity, hypoxia, and serum withdrawal, through a process that requires neuronal expression of VEGFR2 [122, 186–188]. Similarly, VEGFA promotes the survival of migrating neuroendocrine cells through neuronal- and not endothelial-expressed NRP-1 [131]. Remarkably, VEGFA administered at neuroprotective doses in an amyotrophic lateral sclerosis (ALS) rat model preserved motor neurons without significant vascular effects [189]. In the ischemic brain, VEGFA-mediated neuroprotective effects preceded any signs of angiogenesis or neurogenesis, and in traumatic brain injury models, VEGFA reduced hippocampal neuronal apoptosis without influencing neuronal proliferation [167, 176]. The molecular mechanisms underlying VEGFA-mediated neuroprotection are not fully understood, but involve phosphoinositide 3-kinase (PI3K)/Akt and mitogen-activated protein kinase (MAPK) pathways, phosphorylation of the voltage-gated potassium channel Kv1.2, as well as reduction of ischemia-induced Ca^2+^ influx through inhibition of voltage-gated channels [41, 186–188, 190–194].

PlGF’s role in neuroprotection is more controversial as it appears to be more complex and context-dependent. On one hand, PlGF induces angiogenesis, reduces apoptosis and infarct volume, improves behavioral performance in a rat stroke model and protects mitochondrial function in cultured neurons after oxygen–glucose deprivation [195, 196]. PlGF, partially through activation of MEK and PI3K pathways and suppression of caspases, also promotes survival of retinal neurons in response to oxygen–glucose deprivation or light irradiation in vitro [197]. On the other hand, PlGF failed to protect cultured cortical neurons from ischemic death [122]. Further, in an in vivo mouse model of light-induced retinal damage, PlGF exacerbated neuronal death by promoting blood–retinal barrier breakdown, and anti-PlGF antibody treatments were protective [198]. In sum, these contradictory findings underscore the need for targeted investigations to resolve the dual roles of PlGF and define its therapeutic applicability.

VEGFB is emerging as a potent neuroprotective factor with direct effects on neurons. It promotes survival of several cell types in response to toxic stimuli such as cultured DRG, retinal, hippocampal, and trigeminal ganglia neurons [132, 134, 135, 199, 200]. VEGFB also has neuroprotective effects in several pathological conditions, including cerebral ischemia and neurodegeneration, which have been recently reviewed [201]. In brief, VEGFB reduced hypoxia-induced cell death of mouse cortical neurons in culture and mice lacking VEGFB were more susceptible to ischemia-induced brain damage [60]. Moreover, VEGFB was neuroprotective in mouse models of stroke and ocular neurodegenerative disorders [134]. VEGFB appears to be valuable for the potential treatment of motor disorders. VEGFB deficiency worsens motor neuron degeneration in an ALS model, while supplementation of VEGFB protects cultured motor neurons from growth factor withdrawal-induced cell death [202]. Furthermore, delivery of recombinant VEGFB via osmotic pumps into the brain ventricles of ALS rodent model protects brainstem motor neurons and enhanced survival and relieved diseases symptoms [202]. Administration of VEGFB was also neuroprotective in cultures of dopaminergic neurons from Parkinson’s disease (PD) rat models, as well as in in vivo PD rat models [203–205]. Finally, a recent neuroprotective effect of VEGFB in cerebellar Purkinje cells in a mouse model of childhood-onset neurodegeneration with cerebellar atrophy (CONDCA) was also reported [206]. Notably, in contrast to VEGFA, VEGFB’s neuroprotective effects occur independently of angiogenesis, mediated instead by neuronal VEGFR1 [134, 202, 207]. This pathway activates ERK1/2 and Akt signaling and upregulates antioxidative and antiapoptotic mechanisms [134, 135, 200, 203]. Furthermore, in an in vitro cell line model for PD, VEGFB-VEGFR1 upregulates genes involved in mitochondrial fatty acid metabolism and protects against stress-induced mitochondrial membrane potential breakdown in DRG cells, indicating that one of the mechanisms of VEGFB-induced neuroprotection is through prevention of mitochondrial damage [201, 203, 207]. These findings highlight VEGFB’s potential as a non-angiogenic neuroprotective therapy with broad applicability.

VEGFC has demonstrated neuroprotective effects in PD and stroke models. In a rat unilateral 6-OHDA model of PD, VEGFC promoted dopaminergic neuron survival both in vitro and in vivo through striatal ERK1/2 activation [208]. Additional neuroprotective effects may arise indirectly through angiogenesis and glial cell activation [208]. In a stroke mouse model, intrathecal or intracerebroventricular prophylactic delivery of viral-encoded VEGFC increased lymphatic drainage and, thus, promotes neuroprotective signaling and neurological recovery [209, 210].

VEGFD also showed neuroprotective properties with most evidence linked to preservation of neuronal integrity in pathologies linked to glutamate overload and excitotoxicity. Indeed, mouse VEGFD neuronal expression quickly decreased following excitotoxicity in the adult retina or in the cortex after stroke [139, 211]. Intriguingly, expression of the VEGFD receptor VEGFR3 was induced after a stroke event in mice, possibly indicative of some compensatory mechanisms [139, 212]. VEGFD, through direct activation of VEGFR3 on retinal ganglion cells (RGC), protected against excitotoxicity-induced RGC death and preserved their functions [211]. VEGFD delivery in a stroke model successfully protected basal dendrites of pyramidal neurons in layer 2/3 of motor cortex from stroke-induced damage. This resulted in a smaller infarct area and better functional motor recovery of the mice post stroke. The neuroprotection appeared to be mediated by a direct effect on neurons as no effects on superficial pial vessels or capillaries were detected [139]. Intriguingly, these effects are specific to VEGFD, as VEGFC and VEGFA fail to replicate them [139]. Moreover, the structural and functional protection could still be achieved via nasally delivered recombinant VEGFD or VEGFD mimetic peptide and further underscores its translational potential [139].

Despite promising preclinical results, several challenges must be addressed to realize VEGFs’ therapeutic potential. Comparative studies across VEGF family members and diseases will further clarify their therapeutic niches and further research is also needed to clarify some conflicting findings. In example, the dual roles of VEGFs, such as PlGF’s protective and damaging effects, require in depth-specific studies to really define the mechanism of action. Further, future delivery methods must ensure targeted action to minimize off-target effects, especially in angiogenesis-independent applications. Advances in viral vectors, mimetic peptides, and isoform-specific VEGFs provide promising avenues for achieving this. Indeed, in the field dealing with retina pathologies, viral mediated deliveries, which can be cell-specific due to tropism and molecular biology engineering, are a fast-developing reality.

## Conclusion and outlook

In conclusion, VEGFs family members, traditionally known for their roles in blood and lymphatics vessel formation and maintenance, are increasingly recognized for their pivotal roles in the nervous system for functions that extend beyond vascular effects. Further, many of these effects derive from direct actions on neurons. Intriguingly, the presence of VEGF homologs in invertebrates with only rudimentary vascular systems suggests a possible origin of VEGFs in the nervous system than in the vascular context [265]. VEGFs modulate key aspects of neuronal development such as neurogenesis and migration. Further, they support neuronal resilience against injury and neurodegeneration. VEGFs significantly modulate synaptic functions by promoting synaptogenesis, enhancing synaptic stability, influencing neurotransmitter release and receptor expression at the synapse, fine-tuning synaptic strength and responsiveness. Finally, these secreted factors impact many key aspects of neuronal architecture. Future research on VEGFs may focus and expand their potential to reshape and repair neuronal architecture, especially in contexts of neurodegeneration and neural injury. By exploring how VEGFs, including distinct isoforms and contexts, specifically influence axonal growth, dendritic branching, and synaptic formation, scientists could harness these molecules to support neural connectivity and circuit integrity in the myriads of diseases where neural architecture is compromised. Treatments targeting VEGF pathways in vascular cells are being developed or already under use for cancer and eye diseases. However, since it is now clear that VEGFs play multiple key roles in the nervous system, it is thus also crucial to consider their potential long-term effects, as such therapies may unintentionally impact neuronal functions.

## Data Availability

Not applicable.

## Abbreviations

6-OHDA: 6-Hydroxydopamine
AD: Alzheimer’s disease
ALS: Amyotrophic lateral sclerosis
AMPAR: α-Amino-3-hydroxy-5-methyl-4-isoxazolepropionic acid receptor
ATF2: Activating transcription factor 2
CaMKII: Calcium–calmodulin-activated kinase
CONDCA: Childhood-onset neurodegeneration with cerebellar atrophy
CREB: Camp response element-binding protein
DRG: Dorsal root ganglion
ECM: Extracellular matrix
EGF: Epidermal growth factor
EGFL7: Epidermal growth factor-like protein 7
EPSC: Excitatory postsynaptic currents
EPSP: Excitatory postsynaptic potential
ERK: Extracellular signal-regulated protein kinase
FGF-2: Fibroblast growth factor 2
Flk1: Fetal liver kinase 1
Flt1: Fms-like tyrosine kinase 1
Flt4: Fms-like tyrosine kinase 4
GSKα/β: Glycogen synthase kinase 3 α/β
HDAC4: Histone Deacetylase 4
hiPSC: Human induced pluripotent stem cell
HSPG: Heparan sulfate proteoglycan
IPSP: Inhibitory postsynaptic potential
JNK: C-Jun N-terminal kinase
Kdr: Kinase insert domain-containing receptor
L-VACC: Voltage-gated Na + channels
MAP-2: Microtubule-associated protein-2
MAPK: Mitogen-activated protein kinase
MCAO: Middle cerebral artery occlusion
mEPSC: Miniature excitatory postsynaptic currents
MKK4: Mitogen-activated protein kinase kinase 4
MSK1: Mitogen stress-activated kinase 1
NMDAR: N-methyl-D-aspartate receptor
NRP-1: Neuropilin-1
NRP-2: Neuropilin-2
NSC: Neuronal stem cell
PD: Parkinson’s disease
PDGF: Platelet-derived growth factor
PI3K: Phosphoinositide 3-kinase
PKC: Protein kinase C
PLCγ: Phospholipase Cγ-inositol 1,4,5-triphosphate
PlGF: Placental growth factor
PNS: Peripheral nervous system
RGC: Retinal ganglion cell
ROK: Rho-associated protein kinase
RTK: Receptor tyrosine kinase
SFK: Src family kinase
SGZ: Subgranular zone
SOD1: Superoxide dismutase 1
STEP: Striatal-enriched protein tyrosine phosphatase
SVZ: Subventricular zone
VEGF: Vascular endothelial growth factor
VEGFR: Vascular endothelial growth factor receptor
VHD: VEGF homology domain
